# Optimizing split-fertilizer applications for enhanced maize yield and nutrient use efficiency in Nigeria's Middle-belt

**DOI:** 10.1016/j.heliyon.2024.e37747

**Published:** 2024-09-14

**Authors:** Kehinde Ojeniyi, Chirinda Ngonidzashe, Krishna Devkota, Donald Madukwe

**Affiliations:** a Mohammed VI Polytechnic University, College of Agriculture and Environmental Sciences (CAES), Agricultural Innovations and Technology Transfer Centre (AITTC), Benguerir, Morocco; b International Center for Agricultural Research in Dry Areas (ICARDA), Rabat, Morocco; c Agronomy Department, OCP Africa, Abuja, Nigeria

**Keywords:** Fertilizer optimization, Sustainable crop management, Site-specific nutrient management, Maize yield improvement, Maize fertilization

## Abstract

Inadequate and imbalanced fertilizer application is a significant barrier to achieving higher maize yields in Nigeria's Middle Belt. This study hypothesized that optimizing fertilizer types and application rates, particularly through split applications of straight fertilizers, can significantly enhance maize yield and nutrient use efficiency compared to conventional NPK blends and farmer's practices. This experiment evaluated the effects of optimizing types and amounts of fertilizer on maize growth and yield, soil characteristics, and nutrient use efficiencies in the mid-belt region of Nigeria. A field experiment was conducted at two locations using a randomized complete block design with four replications. The treatments included national and regional fertilizer recommendations, applied as NPK blends and straight fertilizers, along with a farmer's practice and control. Soil samples were collected before and after the experiment, and data on yield, yield attributes, grain, and leaf samples, were collected for analysis. The results showed that split applications of straight fertilizers increased grain yield by 22 %–46 %, achieving yields ranging from 2.37 to 3.08 t ha^−1^, compared to yields from NPK blends. Nitrogen uptake efficiency improved by up to 52 %, while potassium uptake exceeded 100 % in certain treatments. Despite higher input costs, split applications yielded gross margins up to 35 % greater than those obtained with NPK blends, underscoring their economic viability. Split application of regional recommendation of 119:38:20 kg ha^−1^ of N, P, and K from straight fertilizer shows higher yields and better nutrient efficiency than NPK blends, proving effective for optimum maize production in the region. No significant changes in soil physio-chemical properties, suggesting that long-term studies are needed to fully understand the impact of fertilizer practices on soil health**.** These findings strongly support the adoption of site-specific nutrient management strategies, particularly the use of straight fertilizers in split applications, to maximize maize production in Nigeria's Middle-Belt.

## Highlights

• Straight fertilizer application results in decreased plant height and reduced biomass yield in maize.
• Straight fertilizer applications produce higher maize yields compared to corresponding NPK blends.
• Application of straight fertilizer enhances nutrient and agronomic efficiency in maize.

## 1 Introduction

Maize (*Zea mays*. L) plays a crucial role as a staple cereal crop in Nigeria, driven by the country's growing population, expanding poultry sector, and increasing demand from the food processing industry. Globally, Nigeria stands as the 12th largest producer of maize and holds the 2nd largest production rank on the African continent [1]. From 1971 to 2020, Nigeria's maize production significantly increased, rising from 931,000 tons to 10 million tons, with an average annual growth rate of 7.29 %. In 2020 alone, Nigeria produced 10 million tons of maize (Knoema, 2020). Despite these achievements, the production of cereals in Nigeria still falls short of meeting domestic consumption needs [2]. With the population and food demand projected to increase by 50 % by 2050 (Worldometer, 2023), the potential maize yields in Nigeria, estimated between 9 and 12 t ha⁻^1^ (GYGA, 2017 [3]), are far from being realized. The average yield remains low at 1.3 to 2 t ha⁻^1^, resulting in annual production of approximately 11 million metric tons ([4] [5]; [1]), which is insufficient to meet the estimated annual demand of 12–15 million metric tons [1]. Bridging this 2 to 4 million metric ton gap is critical to meeting rising food needs and supporting export potential, underscoring the urgency of developing cost-effective strategies to enhance maize production in Nigeria.

The Nigerian government has implemented several initiatives to boost maize production, including fertilizer subsidies [6]. However, these efforts have had limited success in raising maize yields due to challenges such as poor soil quality, improper fertilizer application methods, and a lack of tailored fertilizer rates to meet specific crop needs [5]. Farmers commonly apply blended NPK 15:15:15 fertilizers at 300 kg ha^−1^ and urea at 100 kg ha^−1^ (IITA, 2020). Yet, research by Aliyu et al. [7] has revealed varying N:P requirements across three management zones in Nigeria's mid-belt—a key maize producing region—with needs ranging from 89:34:15 to 138:45:21 kg ha^−1^. A major issue contributing to suboptimal nutrient delivery is the outdated technology used by blending companies, leading to uneven fertilizer distribution [8]. In contrast, using straight fertilizers enables more precise nutrient application in terms of rate and timing, thereby improving nutrient absorption and plant uptake. This study examines the impact of applying national and regional fertilizer recommendations—using straight and blended fertilizers—on maize growth, yields, soil properties, and nutrient use efficiencies in Nigeria's mid-belt region.

Research conducted across various regions, including Nigeria, Sub-Saharan Africa, and Asia, has demonstrated that improving fertilizer efficiency can significantly increase crop yields. However, improper use or overapplication of fertilizers can have negative consequences. For instance, Devkota et al. [9,10] found that in the Nawalparasi and Palpa districts of Nepal, implementing balanced nutrient management and best practices resulted in more than a100 % increase in maize yield. Similarly, Mahamood et al. [11] reported a high grain yield of 8.37 t ha^−1^ with a balanced application of N_300_P_50_K_150_S_30_. Vanlauwe et al. [12] emphasized the importance of precise fertilizer application, noting that continuous cultivation with accurate nitrogen use led to significantly improved nitrogen-use efficiency compared to fields that were not consistently cultivated. On the other hand, Meng et al. [13] warned against the excessive use of nitrogen fertilizers, particularly in China, where misunderstandings of the nitrogen uptake-yield relationship have led to overapplication and poorly timed fertilizer use, ultimately disrupting the balance between crop nitrogen needs and supply.

Since the introduction of inorganic fertilizers in Nigeria in 1937 [14], there has been limited research specifically comparing the impact of straight versus blended fertilizers on nutrient uptake in the country. A notable exception is a recent study by Akinbani & Ayeni [15] on cassava in southwestern Nigeria, which examined the effects of different fertilizer types on nutrient uptake and yield. While various studies have investigated the effects of fertilizers on maize yield and nutrient uptake, few have directly compared straight and blended fertilizers. For instance, Tofa et al. [16] explored the interactive effects of nitrogen and phosphorus fertilizers, Ayeni et al. 2012; [17]) studied the impacts of organic, organomineral, and mineral fertilizers, Ogendo et al. [18] focused on fecal fertilizers, and Waniyo et al. [19] examined different manure types. Other studies include Eifediyi et al. [20] on NPK and organomineral fertilizers for jute mallow, Ayoola & Makinde [21] on nitrogen-enriched organic fertilizers, Ayoola [22] on mixed cropping systems, Olowoake et al. [23] on farmyard manure and urea, Nnana et al. [24] on the effects of various manures, and Arubalueze [25] on nitrogen and phosphorus rates.

This study addresses a crucial gap by examining the effects of straight versus blended fertilizers on nutrient efficiency, soil properties, and maize yield in Nigeria's mid-belt region. It introduces an innovative approach by comparing national and regional fertilizer recommendations using straight and blended fertilizers, an area that has been scarcely explored in this region. The research aims to determine how these different fertilizer types influence maize growth, yield, soil properties, and nutrient use efficiencies, ultimately providing a way forward for more effective nutrient management strategies. By integrating an evaluation of farmers' practices with field experiments, the study generates robust data that provides valuable insights for advancing agricultural practices and policy recommendations. The research focuses on critical questions, such as the impact of split applications of straight fertilizer on maize yield, the nutrient utilization efficiency when fertilizers are applied in both blended and straight formulations, and the effectiveness of current fertilizer recommendations in enhancing maize productivity. Through a well-designed field experiment, this study evaluates the effectiveness of different fertilizer application methods in improving soil fertility, assesses yield differences between straight and blended fertilizers, and examines the overall effectiveness of current fertilizer recommendations for maize production in Nigeria's mid-belt.

## 2 Material and methods

### 2.1 Climate and soils of the study sites

The study was conducted at Mandala, Niger State (N 9° 4′ 27.9984, E 7° 29′ 15). Two sites were selected to assess differences in location and treatment efficacy, ensuring the validation and reliability of the findings. Both sites fall within the same agroecological zone, characterized by a single rainy season from April to September and a dry season from December to March, with an average annual rainfall of 1,200 mm and temperatures reaching 36 °C [26]. According to Shehu et al. [27], the soils in this region are slightly alkaline, with low iron (Fe) content and a sandy loam texture, with organic carbon and total nitrogen levels below critical thresholds. Pre-planting soil analysis revealed average pH levels of 6.5 at site 1 and 6.3 at site 2, with both sites showing similar cation exchange capacities (5.0 cmol kg^−1^) and total nitrogen levels around 0.7 %. However, there were differences in exchangeable potassium (K), with site 1 measuring 0.1 cmol kg^−1^ and site 2 at 0.2 cmol kg^−1^. Both sites were deficient in organic matter, and available phosphorus was within critical limits.

### 2.2 Baseline survey for the characterization of maize production systems

To understand farmer's practices related to fertilizer application and soil nutrient management, a baseline survey was conducted in January 2023 among 30 maize farmers during a Life Restoration Program (LRP) meeting at the OCP blending plant in Kaduna. All participants were experienced maize producers. The survey, administered in printed format, included guides to assist with any unclear questions. A structured questionnaire with fixed responses was used to gather data on the types of fertilizers used, application rates, frequency, methods, and resulting grain yields. Additionally, the survey collected information on farmers' market access, demographic characteristics, and overall production practices. The goal was to assess current practices, identify areas for improvement, and use this information to design field experiments that align with farmers' needs and determine the best ways to support them.

### 2.3 Experimental design and treatments

In the 2023 farming season, an on-station experiment was conducted across two locations in Mandala, Niger State. The experiment was set up with two blocks, each containing treatments replicated three times. The plots, measuring 3m × 3m were arranged using a completely randomized block design (RCBD). The experimental setup includes six treatments in total:

oT1 (CTRL): No fertilizer application
o T2 (FP): Farmer practice in terms of rate and method of application
o T3 (NR-NPK): National Recommendation applied as NPK blend
o T4 (NR-Split): National Recommendation applied in straight form
o T5 (RR-NPK): Regional Recommendation applied as NPK blend
o T6 (RR-split): Regional Recommendation applied in straight form.

### 2.4 Crop management practices

Before the experiment, maize was grown at the first site and tomatoes at the second site. The land was cleared with herbicide (paraquat) at 16.7 ml L^−1^ of water, manually plowed after three days, and planted with the Super-king F1 maize variety four days later with a spacing of 70 cm × 30 cm (48,000 plants ha^−1^). The planting was done on March 2, 2023 and harvested on May 12, 2023. Weeding was done manually at two and five week after sowing (WAS). Drip irrigation was provided at 1.3 L h^−1^ twice daily until the rainy season began.

The basal fertilizer was applied one WAS by spot application at 2 cm soil depths, except for the farmer's practice (FP), where it was applied at two WAS on the soil surface. Birds dig out some of the planted seeds, so replanting was done at one WAS, but the fertilizer was applied as a base (beneath the seed) instead of waiting for after a week again; this was done to ensure uniformity of treatment application. Although manual weeding was adopted for the experiment, the herbicide cost was estimated as shown in supplementary information (SI) SITable 1 was to estimate the production cost on a large scale.

**Table 1:** Fertilizer application per treatment.

Trts	Rate (kg ha^−1^)	7 DAS	32 DAS	65 DAS
		Rate (kg ha^−1^)		
Control	0	0	0	0
FP	120:30:30	200 kg NPK	200 kg Urea	0
NR-NPK	91:45:45	300 kg NPK	100 Urea	0
NR-split	91:45:45	49 + 168+38 Urea/SSP/MOP	101 + 56+23 Urea/SSP/MOP	49 + 0+15 Urea/SSP/MOP
RR-NPK	119:38:20	253 kg NPK	176 kg Urea	0
RR-split	119:38:20	65 + 143+16 Urea/SSP/MOP	129 + 47+10 Urea/SSP/MOP	65 + 0+7 Urea/SSP/MOP
% of Applications for split		**25%N, 75%P 50%K**	**50%N, 25%P 30%K**	**25%N, 0%P 20%K**

DAS = Days after sowing, WAS= Week after sowing, FP= Farmer's practice, NR= National recommendations, RR= Regional recommendations, SSP= Single supper phosphate, MOP = Muriate of potash, NPK=NPK Blend.

∗∗ Fertilizer was applied to FP at 2WAS and top-dressed at 4WAS.

### 2.5 Fertilizer application

Considering the dynamic nature of nitrogen in the soil (mobility, leaching, volatilization), as presented in Table 1, the application rates were split three times for T4 and T6, 25 % at basal, 50 % at V10 (when the plant has 10 leaves), approximately around 30–32 days after sowing (DAS) [28]; and 25 % during VT/R1 stage (just after the vegetative stage, around 20–24 leave) at about 65 DAS [29]. P and K are applied at 75 % and 50 % at planting, 25 % and 30 % at the first top dress, and 0 % and 20 % at the second top dress, respectively, refer to Table 1. The fertilizer application schedule was designed to evaluate various fertilizer management practices and their impact on maize yield and nutrient efficiency.

● Farmer's Practice (T2): The NPK (15:15:15) was applied at two WAS and top-dressed with urea at 4 WAS according to the farmers' usual practice, as determined from the survey. This was done to assess the yield potential under the farmer's current management practice.
● NPK Blends (T3 and T5): The NPK (15:15:15) was applied at one WAS and top dress with urea at four WAS following Kamara et al. [30] recommendations for NPK blend application, with two applications: at/before seven DAS and top-dressed with urea at sixty DAS Table 1. The aim was to evaluate the potential yield achievable with recommended practices.
● Split Applications (T4 and T6): The straight fertilizers were sourced from Urea, single super phosphate (SSP), and muriate of potash (MOP). The fertilizer was applied in split doses at seven DAS, thirty DAS, and sixty DAS Table 1, aligning with the crop's nutrient requirements throughout the growing season. The aim was to assess the yield and efficiency of split fertilizer application using straight fertilizers.
● Application Methods**:** The surface-spot application method was used for the FP, as indicated by the survey findings (SI Table 2). For the other treatments (T3, T4, T5, and T6), the fertilizer was applied 2 cm below the soil surface following the recommendations of APNI [31].

**Table 2:** Physicochemical properties of the soil before and after harvest as affected by different treatments in mid-region Nigeria.

Treatments	Before planting		Control		FP		NR-NPK		NR-SPLIT		RR-NPK		RR-SPLIT		Mean	
	Site 1	Site 2	Site1	Site 2	Site 1	Site 2	Site 1	Site 2	Site 1	Site 2	Site 1	Site 2	Site 1	Site 2	Site 1	Site 2
pH	6.5	6.3	6.70	6.50	6.60	6.77	6.70	6.73	6.730	6.97	6.60	7.00	6.80	6.73	6.7	6.9
CEC (cmol kg^−1^)	5.0	5.4	5.27	5.63	4.97	5.43	4.97	5.13	5.17	5.10	5.43	5.40	5.37	5.50	5.2	5.4
Total N (%)	0.07	0.08	0.07	0.07	0.07	0.07	0.07	0.06	0.07	0.06	0.07	0.07	0.07	0.07	0.07	0.07
Available P (mg kg^−1^)	30.0	27.5	26.7	26.7	26.7	36.7	33.3	26.7	26.7	33.3	30.0	40.0	40.0	33.3	30.6	32.78
Organic Matter (g kg^−1^)	1.65	2.3	1.70	1.83	1.70	1.83	1.77	1.67	1.80	1.57	1.73	1.50	1.80	1.93	1.75	1.72
Exchangeable K^+^ (cmol kg^−1^)	0.1	0.2	0.12	0.17	0.11	0.14	0.12	0.12	0.12	0.14	0.18	0.18	0.20	0.20	0.2	0.2
Exchangeable Ca^2+^ (cmol kg^−1^)	3.8	3.8	4.00	4.50	3.83	4.33	3.83	3.83	3.83	4.17	4.00	4.50	3.83	4.33	3.9	4.3
Exchangeable Mg^+^(cmol kg^−1^)	0.7	0.8	0.67	0.78	0.67	0.78	0.72	0.72	0.72	0.72	0.75	0.83	0.86	0.78	0.7	0.8
Texture	Loam sand	Loam sand	_	_	_	_	_	_	_	_	_	_	_	_	_	_

FP= Farmer's practice, NR= National recommendations, RR= Regional recommendations, NPK=NPK Blend, CEC= Cation exchange capacity, N = Nitrogen, P= Phosphorus, K = Potassium, Ca = Calcium, Mg = Magnesium.

### 2.6 Measured observations

#### 2.6.1 Maize phenological data, yield, and yield attributes

The total grain yield at less than 15 % moisture content was measured from each plot using a scale, while the 100-grain weight was measured using a digital scale. The number of cob/plants was taken from the four tagged plants within the net plots. The plant height was measured in (cm) from the tag plants within each plot at 3, 6, 9 WAS, and at harvest. The biomass yield was measured for each plot at the end of the experiment using a weighing scale. Days to 50 % tasselling and days to 50 % heading were recorded. The harvest index (HI) was calculated by dividing the grain yield by the biomass weight. The data for fresh cob yield was also taken because the economic analysis was based on the cost per kilogram of fresh cob.

#### 2.6.2 Soil and plant analysis

Four soil samples (two composite samples per site) were collected before planting, and 36 samples (one composite sample per plot × 6 treatments × 3 replications × 2 sites) were collected after harvest at a depth of 20 cm using an auger. The samples were stored in transparent Ziplock bags and air-dried for one week. The following parameters were analyzed at Cropnut Lab using a mid-infrared (MIR) scanner [32]: pH, available phosphorus (P), exchangeable potassium (K), total nitrogen, organic matter, cation exchange capacity (CEC), calcium (Ca), and magnesium (Mg). Additionally, plant samples (grain and leaves) were collected at harvest from tagged plants in the net plot for nitrogen (N), phosphorus (P), and potassium (K) analysis. The plant samples were air-dried to a constant weight and then ground into powder for nutrient analysis.

#### 2.6.3 Plant nitrogen content

Nitrogen was analyzed by the Kjeldahl method as described by Sáez-Plaza et al. [33] One gram of the sample was measured into a 250 ml Kjeldahl tube. Copper (II) sulfate (0.6 g) was added as a catalyst, 7–10 g of potassium sulfate was added to increase the boiling point, and 0.2 g of zinc granules were added to reduce bumping. Concentrated sulfuric acid (10 ml, 98 %) was added, and the mixture was digested at 370 °C for 15–20 min. After cooling, the digest was diluted with distilled water and transferred to a 500 ml volumetric flask, which was then filled to the mark. From this solution, 50 ml was measured into a 250 ml Kjeldahl flask. 5 ml of 50 % sodium hydroxide solution and 100 ml of distilled water were added. The receiving flask contained 100 ml of 4 % boric acid solution and 4–5 drops of methyl red indicator. The solution was distilled for approximately 13 min, and the distillate was collected and titrated against hydrochloric acid until the first color changed. The titre value was recorded and the percentage of nitrogen was calculated as follows:

$$\%\;N=\frac{Titre\;value*Normality\;of\;acid*make\;up\;volume\left(ml\right)*molecular\;weigth*100}{Sample\;weigth\;\left(g\right)*100*volume\;used\;\left(ml\right)}$$

#### 2.6.4 Available phosphorus content in the grain and leave

Phosphorus was analyzed following the method described by Maher & Woo [34]. One gram of the sample was placed in a 250 ml flask, followed by the addition of 10 ml of nitric acid. The mixture was heated on a hot plate until frothing occurred, after which perchloric acid was added, and digestion continued until white precipitates formed. The digest was then filtered using a 0.25-μm Whatman No. 41 filter paper. The filtrate was collected in a 100 ml conical flask, partially filled with distilled water, and then brought to volume with additional distilled water. From this solution, 5 ml was transferred to another 100 ml conical flask, and 10 ml of molybdate vanadate solution was added to precipitate the phosphorus as a phosphomolybdate complex. The phosphorus content was then measured using a Spectroquant Prove 300 instrument. To calculate the available P, the following formula was used:

$$\%\;P=\frac{Absorbent\;different*Factor*Make\;up\;volume\left(ml\right)*100}{Sample\;weigth\;\left(g\right)*100*Volume\;used\;\left(ml\right)*\%\;Dry\;matter/100}$$

here, the absorbent difference is calculated as the difference between the sample and the blank solution.

#### 2.6.5 Exchangeable soil potassium content

The method described by Thomas et al. [35] was used to analyze potassium. One gram of the sample was measured into a 250 ml conical flask, then 10 ml of nitric acid was added. The mixture was digested until it reached a frosting or brown color. After cooling slightly, 10 ml of perchloric acid was added, continuing digestion until the sample turned white. The resulting filtrate was filtered into a 250 ml flask and filled to the mark (volume made up). From the filtrate, 1 ml was pipetted into another 250 ml flask, to which 2 ml of a masking reagent (interference suppressor) was added, filling the flask to the mark. Potassium concentration was measured at a wavelength of 7665 nm using an Atomic Absorption Spectrometer ICE 3000.

To calculate the percentage of potassium (%K), the following formula was used:

$$Exchangeable\;K=\frac{Concentration\;\left(ppm\right)*Make\;up\;volume\left(ml\right)*Dilutionfactor}{Sample\;weigth\;\left(g\right)*\%\;dry\frac{matter}{100}}$$

#### 2.6.6 Plant dry matter accumulation

The weight of an empty Petri dish was recorded. Then, 2 g of the dry sample of the seed and plant were separately measured and placed into the Petri dish, and the weight was re-recorded. The sample was then oven-dried at 60 °C for 3 h. Afterward, it was placed into a desiccator to cool down, and the weight was measured again. The percentage of dry matter was calculated using the formula:

$$\%\;Dry\;matter=\frac{Weigth\;\left(dry\;sample+container\right)-Weigth\;\left(empty\;container\right)}{Weigth\;\left(wet\;sample+container\right)-Weigth\;\left(empty\;container\right)}\;*\;100$$

All units in gram.

### 2.7 Nutrient use efficiencies and uptakes

#### 2.7.1 Agronomic use efficiency

The agronomic efficiency was calculated as described by APNI [31]. Agronomic efficiency AE is calculated as the total yield produced for each quantity of fertilizer applied.

$$AE=\frac{Y-Y0}{F}$$

where: Y = crop yield with fertilizer applied in kg ha^−1^, Y0 = crop yield with no fertilizer applied in kg ha^−1^. F = amount of fertilizer nutrient applied in kg ha^−1^.

#### 2.7.2 Estimation of nutrient efficiency indices

The efficiency of the applied nutrients was determined under the following parameters: N uptake efficiency, N utilization efficiency, N use efficiency, and N harvest index, as discussed by Chander et al. [36].

#### 2.7.3 Nutrient uptake efficiency

Nutrient uptake efficiency was derived from total plant uptake divided by the total nutrient applied for each nutrient using the following formulae.

$${NUpE\;(kg\;kg}^{-1})=\frac{Total\;nutrient\;uptake}{N\;supplied\;}$$

Total nutrient uptake was estimated by multiplying the dry weight of the sample parts by nutrient concentration in the grain and leave and then summing up. The amount of nutrients supplied is the sum of nutrients applied as fertilizer and total nutrient uptake in the control.

#### 2.7.4 Nutrient utilization efficiency

Utilization efficiency was determined by dividing grain yield by total plant N uptake.

$${NUtE\;(kg\;grain\;kg}^{-1}N)=\frac{Grain\;yield}{Total\;nutrient\;uptake}$$

#### 2.7.5 Nutrient use efficiency

NUE was determined by dividing grain yield by nutrients supplied.

$${NUE\;(kg\;grain\;kg}^{-1}N)=\frac{Grain\;yield}{Nutrient\;supplied}$$

#### 2.7.6 Nutrient harvest index

The Harvest Index was determined by dividing the total grain uptake by the total plant uptake and multiplying by 100. The total grain uptake was determined by multiplying the dry weight of the grain by nutrient concentration.

$$HI\;\left(\%\right)=\frac{Grain\;uptake}{Total\;nutrient\;uptake}$$

### 2.8 Economic analysis

Following the method described by Devkota et al. [37], the economic analysis involved calculating total variable costs (TVC), gross margin (GM), gross revenue (GR), and the benefit-to-cost ratio (B:C ratio). TVC was determined assuming the farmer owns the land, by calculating the unit price for all inputs and production costs (as detailed in SI Table 1). Gross revenue (GR) was obtained by summing up the total value of the grain and stover. Gross margin (GM) was then calculated by subtracting TVC from GR, while the B:C ratio was determined by dividing GR by TVC. Additionally, the percentage return on investment (ROI) was estimated.

### 2.9 Statistical analysis

The data analysis was performed using R programming software, version 4.2. Descriptive statistical methods were employed to explore the survey data. To assess the variance among treatments, an analysis of variance (ANOVA) was conducted. The treatment mean differences were analyzed using the least significant difference (LSD) at 5 % level of significance. Before conducting the ANOVA, the normality of the data distribution under different fertilizations was examined for yield using the Shapiro-Wilk test. Conformity of the homogeneity of variance was also performed using Bartlet's test [38]. The normality and homogeneity assumptions of ANOVA were met with non-significant p-values so there was no need for data transformation. The combined use of descriptive statistics, ANOVA, and the Duncan test enabled a comprehensive investigation of the survey data, identifying significant differences among groups and a detailed examination of the results.

## 3 Results

### 3.1 Variation in physicochemical soil properties

The comparison of physicochemical properties before planting and after harvest at Site 1 and Site 2 under different treatments (Table 2) indicates no significant differences. Both sites experienced minor increases in pH. Site 1 pH rose from 6.5 to an average of 6.7 and in site 2 it increased from 6.3 to 6.9. Exchangeable potassium (K^+^), calcium (Ca^2+^), and magnesium (Mg^2+^) also increased slightly at both sites. The K^+^ rose from 0.1 to 0.2 cmol kg^−1^ at Site 1 and remained unchanged at Site 2, while Ca^2+^ increased from 3.8 to 3.9 cmol kg^−1^ at Site 1 and from 3.8 to 4.3 cmol kg^−1^ at Site 2. Mg^2+^ remains the same across sites before and after the experiment. CEC remained stable at Site 2 and increased slightly from 5.0 to 5.2 cmol kg^−1^. N levels remained stable around 0.07 % at both sites, with Site 2 showing a minor decrease from 0.08 % to 0.07 %. Organic matter content showed little variation, increasing slightly from 1.65 to 1.75 g kg^−1^ at Site 1 and decreasing from 2.3 to 1.72 g kg^−1^ at Site 2. Available phosphorus (P) increased slightly from 30 mg kg^−1^ to 30.56 mg kg^−1^ at Site 1 and 27.5 mg kg^−1^ to 32.78 mg kg^−1^ at Site 2.

### 3.2 Characterization of the maize production inputs and outputs

The survey outlined in ( SI Table 2) shows that most participants (77 %) fell within the age range of 20–40 years. The educational background of these farmers was mostly at a basic level, with 40 % having completed only primary education and 50 % finishing secondary education. Additionally, nearly all (97 %) had over a decade of experience in farming. In terms of market connections, a vast majority (97 %) of the farmers reported having some market access. However, access to fertilizer inputs was mixed, with 48 % indicating they had access while 44 % did not. Many respondents (88 %) were not informed about the quality of the fertilizer products they used, though a significant portion (87 %) were aware of market information.

The survey also found that the predominant fertilizers used were NPK, urea (straight), and organic fertilizers, with none of the farmers using SSP or MOP. Regarding application methods, a small fraction of farmers practiced broadcasting (27 %), or band application (10 %), but the majority (63 %) preferred spot application, and none used foliar application. Surface application was the norm for 80 % of the farmers, while 20 % buried the fertilizer beneath the soil surface. No respondents reported applying basal fertilizer one week after sowing. However, most (83 %) applied 3–4 bags of NPK (15:15:15) two WAS, and 87 % top-dressed with 4 bags of Urea four WAS. Organic manure was used by 67 % of the respondents.

The survey further indicated that most respondents were smallholder farmers, with 60 % cultivating 1–5 ha, 23 % farming more than 5 ha, and 17 % operating less than 1 ha. All (100 %) depended on rainfed agriculture, with 67 % manually weeding, 20 % using herbicides for weed control, and only 13 % employing machinery. They faced several challenges, including weed and pest problems, limited capital, issues with herders, and theft. From this result, treatment two (farmer's practice) was designed by applying 200 kg of NPK (15:15:15) two WAS and a top-dress with 200 kg of urea at four WAS, applied by spot application on the soil surface.

### 3.3 Plant height, phenology, yield, and yield attributes

#### 3.3.1 Plant height

**Fig. 1:**
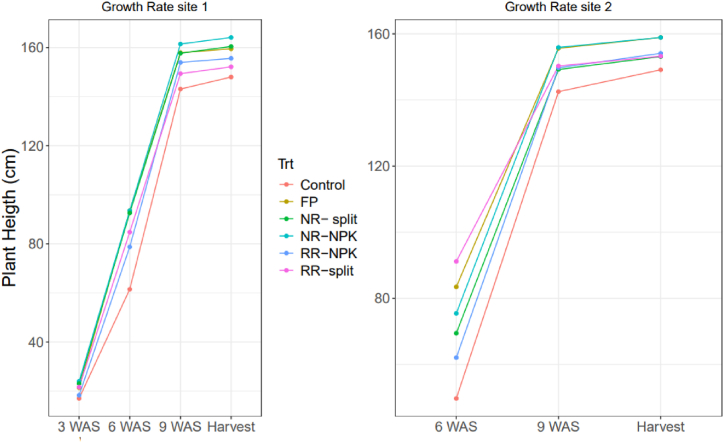
Growth rate for site 1 and 2. WAS= Week after sowing, FP= Farmer's practice, NR= National recommendation, RR= Regional recommendation, NPK= NPK blend.

Figure 1 shows the growth rate. At the first site, the NR-NPK, NR-split, and FP showed comparable growth trends from 3 to 9 WAS, with NR-NPK achieving the greatest height at 161 cm. In contrast, the CTRL exhibited slower growth throughout the growing period, reaching a height of 143 cm. Meanwhile, RR-NPK and RR-split show consistent growth from weeks 6–9. By harvest time, the tallest plants were noted in the NR-NPK at 161 cm, closely followed by FP and NR-split at 158 cm, whereas RR-NPK and RR-split had a vigorous, healthy, shorter plant of 152 cm and 150 cm, respectively. At the second site, initial data collection at 3 weeks was hindered by uneven growth due to rodent damage, yet all treatments exhibited uniform growth from week 6 onwards. By harvest time, RR-NPK, RR-split, and NR-split showed a similar pattern of steady growth. Conversely, FP and NR-NPK had the tallest plants, measuring 158 cm and 159 cm, respectively.

#### 3.3.2 Phenology (days to tasselling and silking) of maize

**Fig. 2:**
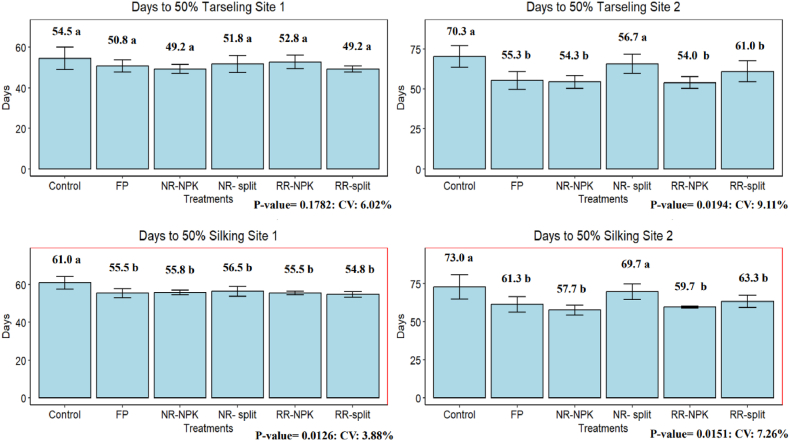
Phenological properties (days to 50 % tarseling and silking) as affected by different treatments. FP= Farmer's practice, NR= National recommendation, RR= Regional recommendation, NPK= NPK blend, CV= Coefficient of variation.

Treatment means comparison revealed that the duration to reach 50 % tasselling (Fig. 2) was not significantly influenced by the treatments at site 1. However, NR-NPK and RR-split reached 50 % tasselling in an average of 49 days, whereas the CTRL took 55 days. At the second site, there was variability among the treatments. The FP, NR-NPK, and RR-NPK reached 50 % tasselling earlier (around 55 days) compared to RR-split and NR-split, which took around 62 days, while the CTRL reached an average of 70 days.

Furthermore, at site 1, the treatment effect on the duration to reach 50 % silking (Fig. 2) showed a similar trend and did not significantly differ, with most fertilized plots reaching 50 % silking at around 55 days, while the CTRL exhibited a statistically significant difference, with an average of 61 days. Nevertheless, at site 2, similar to tasselling, the duration to reach silking also varied among treatments, with the CTRL and NR-split taking more days, 73 and 70 days, respectively. The FP and RR-split took an average of 62 days, and NR-NPK and RR-NPK took around 58 days.

#### 3.3.3 Yield and yield attributes

The total yield of each plot was determined by measuring the weight of the harvested grain at a moisture level below 15 %. This ensures that the yield is accurately represented and not inflated by excess moisture content. The yield result was presented in Table 3. RR-split resulted in the highest grain yield (P < 0.005), with an average of 2.37 t ha^−1^ at site 1, representing a 30 % decrease compared to site 2, which had an average yield of 3.08 t ha^−1^. Grain yield increased in both sites with split applications compared to the NPK blend. However, among the FP, NR-NPK, NR-split, and RR-NPK in site 1, the yields were statistically similar, averaging 1.73, 1.94, 1.98, and 1.80 t ha^−1^, respectively, whereas RR-split exhibited a significantly higher yield of 2.37 t ha^−1^. A similar trend was observed in site 2, except that the RR-NPK and RR-split were statistically similar but different from the national recommendation and FP. A similar trend in grain yield was observed in the cob yield from both sites (Table 3).

**Table 3:** Yield and yield attributes of maize as affected by straight and compound fertilizer treatment in Mid-Belt Nigeria.

Treatments	Grain yield (tha^−1^)		Cob yield (tha^−1^)		No of cob/plant		No of grain/cob		100gw (g)		Biomass yield (tha^−1^)	
	Site 1	Site 2	Site 1	Site 2	Site 1	Site 2	Site 1	Site 2	Site 1	Site 2	Site 1	Site 2
Control	1.20^c^	0.94^c^	8.00^d^	5.20^d^	1^b^	1^a^	461^b^	430^a^	36.25^a^	33.33^a^	4.78^b^	3.57^c^
FP	1.73^bc^	1.62^bc^	11.12^c^	8.80^c^	2^a^	2^a^	527^ab^	409^a^	34.75^a^	37.67^a^	9.90^a^	8.73^a^
NR-NPK	1.94^ab^	2.1^abc^	13.08^bc^	10.63^bc^	2^a^	2^a^	554^ab^	353^a^	31.75^a^	35.00^a^	10.58^a^	7.50^ab^
NR-split	1.98^ab^	1.87^abc^	14.78^ab^	9.67^bc^	2^a^	2^a^	558^a^	465^a^	36.00^a^	37.67^a^	8.20^ab^	4.97^bc^
RR-NPK	1.80^ab^	2.70^ab^	12.50^c^	12.50^ab^	2^a^	2^a^	501^ab^	468^a^	36.50^a^	40.67^a^	7.48^ab^	7.57^ab^
RR-split	2.37^a^	3.08^a^	15.45^a^	14.60^a^	2^a^	2^a^	573^a^	458^a^	36.50^a^	41.00^a^	7.43^ab^	6.50^abc^
CV (%)	20.32	35.54	11.2	18.32	21.24	21.14	11.06	18.58	10.22	11.68	26.34	50.28
Mean	1.84	2.05	12.49	10.23	2	2	529	430	35.29	37.56	8.06	6.47
P-Value	0.0131	0.0504	0.002	0.0019	0.0437	0.4651	0.126	0.5007	0.422	0.2938	0.0206	0.4532

FP= Farmer's practice, NR= National recommendations, RR= Regional recommendations, NPK=NPK Blend, gw = Grain weight, CV= Coefficient of variation.

The number of grains per cob (Table 3) in both NR-split and RR-split was statistically different from other treatments, with average values of 558.75 grains/cob and 573.75 grains/cob, respectively. The treatments in either site did not significantly affect the number of cobs per plant and 100-grain weight. The number of cobs per plant was statistically similar between site 1 and site 2, with mean averages of 2.02 and 2.06, respectively. However, the 100-grain weight was higher (P < 0.005) in site 2 compared to site 1, although statistically similar, with mean values of 35.29 in site 1 and 37.56 in site 2. Across all sites, the biomass yield was statistically similar for all treatments. However, split applications of both fertilizer recommendations generally resulted in lower biomass production than their single application.

#### 3.3.4 Percentage yield difference as affected by fertilizer management

**Fig. 3:**
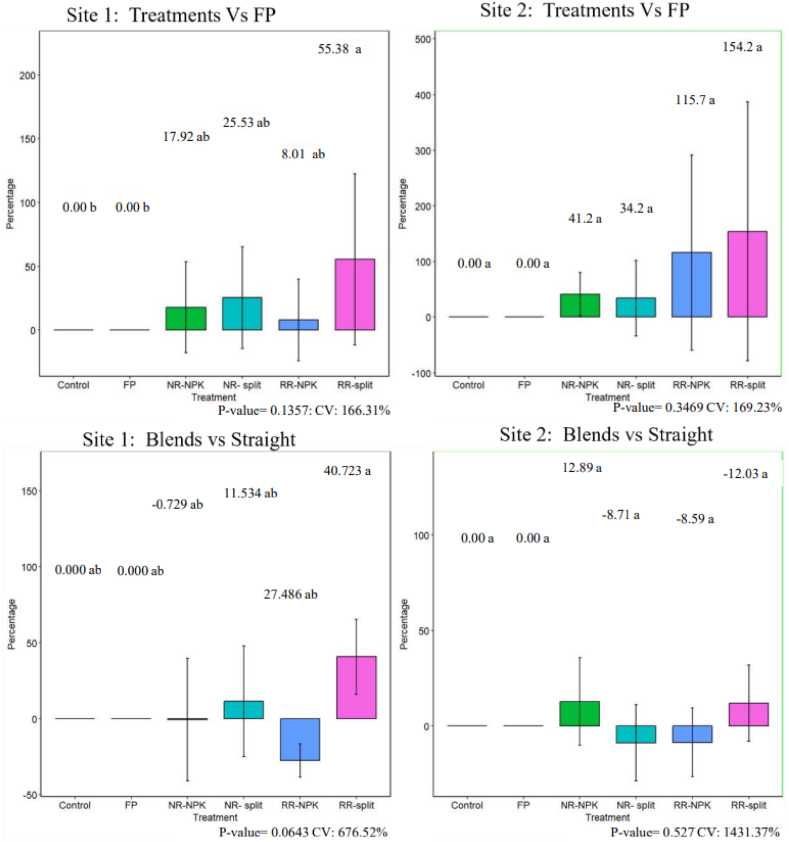
Percentage difference in yield between other treatments and FP and between the NPK blend and straight application for both experimental sites. FP= Farmer's practice, NR= National recommendation, RR= Regional recommendation, NPK= NPK blend, CV= Coefficient of variation.

Fig. 3 illustrates the average percentage difference in yield for each treatment relative to the FP and between the blends and straight fertilizer application across both sites. Among the other treatments vs FP, the data show positive percentage differences, indicating that all treatments outperformed the FP in yield. The RR split produced the most significant yield increase, with a 55.38 % difference at site 1 and an impressive 154.2 % at site 2. On the other hand, in site 1, the NR-NPK and RR-NPK had the smallest yield increases, with percentage differences of 17.92 % and 8.01 %, respectively. In contrast, there was a substantial yield increase from RR in site 2, surpassing the FP by more than 100 %, while NR-NPK and NR-split also showed notable improvements with yield differences of 41.2 % and 34.2 %, respectively ∗() (see Fig. 3).

Both positive and negative results were observed across the sites when comparing the yield differences between split applications and NPK blends ∗(Fig. 3). In site 1, the RR-split and NR-split outperformed their corresponding NPK blends in yield. Specifically, RR-split recorded the largest yield, being 55.38 % greater than RR-NPK, whereas NR-split yielded 11.5 % more than NR-NPK. Conversely, in site 2, while RR-split still surpassed RR-NPK by 12.03 %, NR-split showed a decrease, yielding 8.7 % less than NR-NPK. Overall, treatments involving split applications generally produced higher yields than those with blend applications, though a minor negative deviation was noted for NR-split in site 2.

### 3.4 Nutrient use efficiencies

#### 3.4.1 Nitrogen uptake

The nitrogen effeciency is presented in Table 4. Nitrogen uptake efficiency represents the percentage of applied nitrogen taken up by the crop, measured as kilograms of nitrogen uptake per kilogram of nitrogen applied. In the CTRL, nitrogen uptake is solely attributed to the indigenous nitrogen supply in the soil, resulting in a perfect percentage for both sites. The recorded mean N uptake efficiency of 0.52–0.55 across sites and treatments indicates that the plants absorbed approximately half of the applied nitrogen, except for FP in site 1, which exhibited the lowest uptake efficiency of 0.23.

**Table 4:** Nitrogen efficiency indices on maize production as affected by different treatments in mid-region Nigeria.

Treatments	NUpE (kg N uptake kg^−1^ N applied)		NUtE (kg grain yield kg^−1^ N uptake)		NUE (kg grain yield kg^−1^ N supply)		NHI (%)		Protein content (%)	
	Site 1	Site 2	Site 1	Site 2	Site 1	Site 2	Site 1	Site 2	Site 1	Site 2
Control	1.00^a^	1.00^a^	143^b^	51^d^	0^c^	0^b^	50^ab^	55^b^	9^ab^	16^a^
FP	0.23^c^	0.32^c^	140^a^	117^bc^	45^b^	52^a^	47^ab^	46^c^	8^b^	11^b^
NR-NPK	0.49^b^	0.39^d^	153^ab^	158^ab^	74^a^	61^a^	57^a^	57^ab^	16^a^	12^b^
NR-split	0.49^b^	0.56^b^	156^ab^	100^cd^	76^a^	56^a^	41^b^	56^b^	11^ab^	17^a^
RR-NPK	0.47^b^	0.54^b^	151^b^	136^abc^	71^a^	72^a^	52^ab^	54^b^	14^ab^	16^a^
RR-split	0.47^b^	0.34^e^	155^ab^	174^a^	74^a^	60^a^	46^ab^	62^a^	16^a^	16^a^
CV (%)	9.92	4.2	15.04	22.64	25.13	24.7	12.39	4.94	26.54	11.38
Mean	0.52	0.55	159	123	56	50	49	55	12	15
P-Value	0.0001	0.0001	0.1513	0.0037	0.0004	0.0005	0.1135	0.0013	0.0618	0.0103

FP= Farmer's practice, NR= National recommendations, RR= Regional recommendations, NPK=NPK Blend, NUpE = Nitrogen uptake efficiency, NUtE = Nitrogen utilization efficiency, NUE= Nitrogen use efficiency, NHI= Nitrogen harvest index, CV= Coefficient of variation.

#### 3.4.2 Nitrogen use efficiencies

Nitrogen utilization efficiency reflects the plant's ability to translocate nitrogen into the grain [39]. There was no significant difference (p = 0.1513) among the treatments in site 1, with a mean value of 159.56 kg grain yield kg^−1^ N uptake (Table 4). However, in site 2, treatment RR-split exhibited the highest grain yield per nitrogen uptake, with 174.20 kg grain yield kg^−1^ N uptake. NR-NPK with 158.00 kg grain yield kg^−1^ N uptake showed a 57 % difference in grain yield compared to treatment NR-split, which has 100.90 kg grain yield kg^−1^ N uptake.

Nitrogen use efficiency is determined by the ratio of grain yield to nitrogen supply, expressed as kilograms of grain yield per kilogram of nitrogen applied. The average grain yields at site 1 and site 2 are 56.90 and 50.52, respectively, indicating no significant difference between the two sites. Lower grain yields were observed in FP at both sites, 45.53 and 52.10, respectively. No significant difference in grain yield was observed between the split application and NPK blend.

**Fig. 4:**
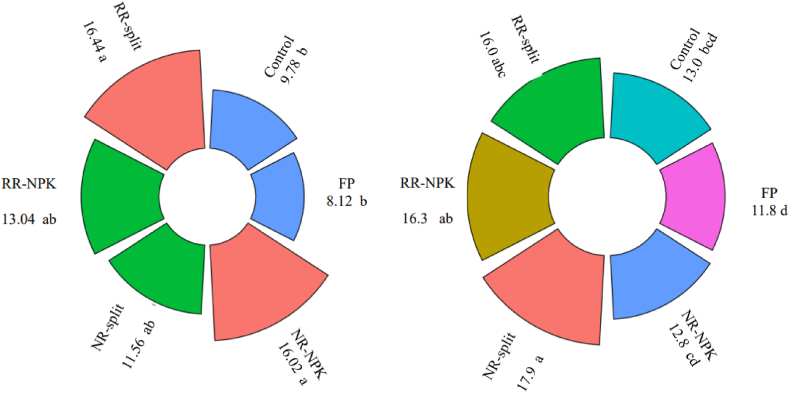
Protein content of the grain for the two sites. FP= Farmer's practice, NR= National recommendation, RR= Regional recommendation, NPK= NPK blend.

The nutrient harvest index represents the proportion of nitrogen accumulated in the grain to the total nitrogen uptake. It also reflects the nutritional quality of the grain due to its association with protein content (Fig. 4). Fertilizer application significantly impacts maize grain quality. While variations exist between NR and RR and split and blend application across two sites, NR and RR generally resulted in higher grain quality compared to the CTRL and FP.

#### 3.4.3 Phosphorus use efficiencies

In both experimental sites, the mean value of phosphorus uptake efficiency was 0.39 and 0.38, respectively (Table 5), indicating challenges in phosphorus uptake due to fixation in the soil associated with tropical soils. The phosphorus use efficiency ranges from 0.15 to 0.38 in site 1 and 0.21 to 0.31 in site 2 (Table 5). Among the treatments, RR-NPK has the highest value of 0.38 in site 1 and 0.31 in site 2. However, there was a higher P uptake among the RR than the NR. Similarly, higher P uptake is also evident between the split and blended applications.

**Table 5:** Phosphorus efficiency indices on maize production as affected by different treatments in mid-region Nigeria.

Treatments	PUpE (kg P uptake kg^−1^ P applied)		PUtE (kg grain yield kg^−1^ P uptake)		PUE (kg grain yield kg^−1^ P supply)		PHI (%)	
	Site 1	Site 2	Site 1	Site 2	Site 1	Site 2	Site 1	Site 2
Control	1.00^a^	1.00^a^	453^b^	178^d^	0^c^	0^c^	42^b^	37^bc^
FP	0.31^c^	0.22^d^	596^b^	492^bc^	182^ab^	105^b^	42^b^	40^ab^
NR-NPK	0.15^e^	0.21^d^	994^a^	590^abc^	150^b^	125^b^	59^a^	38^abc^
NR-split	0.25^d^	0.29^b^	614^b^	386^c^	154^b^	114^b^	36^b^	32^c^
RR-NPK	0.38^b^	0.31^b^	492^b^	614^ab^	188^ab^	191^a^	27^c^	38^abc^
RR-split	0.24^d^	0.26^c^	947^a^	713^a^	232^a^	188^a^	41^b^	44^a^
CV(%)	4.56	4.12	20.67	22.05	23.64	26.94	9.93	9.34
Mean	0.39	0.38	682	495	150	120	41	38
P-Value	0.0001	0.0001	0.0027	0.0017	0.0002	0.0003	0.0001	0.0335

FP= Farmer's practice, NR= National recommendations, RR= Regional recommendations, NPK=NPK Blend, PUpE = Phosphorus uptake efficiency, PUtE = Phosphorus utilization efficiency, PUE= Phosphorus use efficiency, PHI= Phosphorus harvest index, CV= Coefficient of variation.

Furthermore, phosphorus utilization efficiency (PUtE) reflects the plant's ability to translocate phosphorus into the grain. National fertilizer recommendations appeared to utilize P more efficiently as an NPK blend than a split application. In contrast, RR showed a different pattern. RR utilized P better as a split application than as NPK blend.

The RR shows higher P use efficiency in both sites than the NR. The harvest index (PHI), the ratio of phosphorus in grain to total phosphorus uptake, indicates that approximately half of the uptake is accumulated in the grain. Site 1 and 2 show mean values of 41 % and 38 % for PHI, respectively. When applied as NPK, the national recommendation shows a higher PHI of 59 % from site 1, while the RR-split has a PHI of 44 % from site 2.

#### 3.4.4 Potassium use efficiencies

In both sites, potassium uptake efficiency was very high. In some treatments in site 2, the uptake efficiency exceeded 100 %. Despite its availability, the uptake efficiency was higher in split applications compared to NPK blends and farmer practices, as shown in Table 6. Utilization efficiency measures the amount of grain produced from the uptake of potassium. The results from both sites indicate that when applied as an NPK blend, the NR utilizes potassium more efficiently than when applied as a split treatment. RR-split demonstrates the highest potassium utilization efficiency, with yields of 276 kg grain yield kg^−1^ P Uptake in site 1 and 231 kg grain yield kg^−1^ P Uptake in site 2, while FP shows lower efficiency. However, comparing K use efficiency, RR did better, yielding 232 and 357 kg grain yield kg^−1^ K Supply in each site, respectively, compared to the NR. No significant differences exist among the NR when applied as a split treatment or NPK blend. There are no significant differences across the treatments in both sites regarding the harvest index. However, approximately 50 % of the uptake potassium is incorporated into the grain across all sites.

**Table 6:** Potassium use efficiency indices on maize production as affected by different treatments in mid region Nigeria.

Treatments	KUpE (kg K uptake kg^−1^ K applied)		KUtE (kg grain yield kg^−1^ K uptake)		KUE (kg grain yield kg^−1^ K supply)		KHI (%)	
	Site 1	Site 2	Site 1	Site 2	Site 1	Site 2	Site 1	Site 2
Control	1.00a	1.00^d^	151^c^	74^c^	0^c^	0^c^	50^a^	46^b^
FP	0.58c	1.09^c^	207^abc^	146^b^	121^b^	158^b^	48^a^	45^b^
NR-NPK	0.65bc	0.70^e^	231^ab^	177^ab^	150^b^	125^b^	49^a^	44^b^
NR-split	0.70bc	0.72^e^	220^abc^	158^ab^	154^b^	114^b^	45^a^	46^b^
RR-NPK	0.98a	2.19^a^	192^bc^	197^ab^	188^ab^	432^a^	53^a^	50^a^
RR-split	0.84 ab	1.53^b^	276^a^	231^a^	232^a^	357^a^	48^a^	44^b^
CV (%)	12.57	3.28	18.26	24.04	25.08	29.42	9.01	3.87
Mean	0.79	1.2	212	164	140	197	49	46
P-Value	0.0018	0.0001	0.0454	0.0108	0.0004	0.0001	0.3526	0.0083

FP= Farmer's practice, NR= National recommendations, RR= Regional recommendations, NPK=NPK Blend, KUpE = Potassium uptake efficiency, KUtE = Potassium utilization efficiency, KUE= Potassium use efficiency, KHI= Potassium harvest index, CV= Coefficient of variation.

### 3.5 Agronomic efficiency

Agronomic efficiency measures the total yield produced for each quantity of fertilizer applied. Split applications (NR-split and RR-split) generally exhibited higher AE compared to blend applications (NR-NPK and RR-NPK) and FP across all nutrients (N, P, and K). However, the differences between blend and split applications were statistically the same but a bit higher in split applications for both sites. This suggests that while split applications might lead to slightly higher AE for all nutrients, the magnitude of improvement might not be significant. Additionally, RR shows higher AE across treatment and sites than the NR. Higher potassium efficiency was observed among the RR compared to the NR, as indicated in (SI Table 3). However, the two recommendations had no significant differences in site 2 when comparing split applications and NPK blends.

### 3.6 Economics analysis

Assuming the farmer owns the land, the total cost of production (excluding fertilizer) was estimated at $2063.78. The fertilizer cost for each treatment and production contributes to the total variable cost (TVC), as indicated in (Table 7). For economic analysis purposes, the cob yield was used, as sweet corn is sold at the farm gate based on the kilogram yield of cobs, which currently fetches $0.79 per kilogram. The cost of fertilizer is higher in split application than NPK blends, with the split application of NR being 44 % more expensive than applying it as an NPK blend. However, there are minor differences in costs when comparing NR to RR (Table 7). TVC, gross return (GR), and gross margin (GM) are higher in split applications than in NPK blends in both sites. The higher TVC in split applications is primarily due to the increased cost of SSP and MOP fertilizers. Without fertilizer, a return on investment (ROI) of 54 % was recorded in site 1, while site 2 yielded a negative ROI. Interestingly, (FP) provided 187 % and 106 % ROI for sites 1 and 2, respectively. However, significant benefits are observed when fertilizer is applied. NR-NPK yielded an ROI of 248 %, while NR-split offered an ROI of 284 % in site 1. On the other hand, in site 2, NR-NPK resulted in a higher ROI of 164 % compared to 117 % for NR-split. RR-split demonstrates a higher ROI of 311 % and 283 % in sites 1 and 2, respectively, compared to RR-NPK and NR.

**Table 7:** Assessment of the economic benefit of using NPK blend and split fertilizer on maize production (sweet corn) in mid-belt Nigeria.

Treatments	Fertilizer cost ($/ha)	Average cob yield (kg ha^−1^)		TVC ($)	Gross revenue ($)		Gross margin (%)		Benefit-cost ratio		ROI (%)	
		Site 1	Site 2		Site 1	Site 2	Site 1	Site 2	Site 1	Site 2	Site 1	Site 2
Control	–	8,000	5,200	2,063	6,320	4,108	67	50	3.1	2.0	54	−2
FP	200.79	11,120	8,800	2,264	8,784	6,952	74	67	3.9	3.1	187	106
NR-NPK	236.22	13,080	10,630	2,300	10,333	8,397	78	73	4.5	3.7	248	164
NR- split	340.94	14,780	9,670	2,404	11,676	7,639	79	69	4.9	3.2	24	117
RR-NPK	245.14	12,500	12,500	2,308	9,875	9,875	77	77	4.3	4.3	226	226
RR-split	314.96	15,450	14,600	2,378	12,205	11,534	81	79	5.1	4.8	311	283

FP= Farmer's practice, NR= National recommendations, RR= Regional recommendations, NPK=NPK Blend, TVC = Total variable cost, ROI= Return of investment, SSP= Single supper phosphate, MOP = Muriate of potash.

∗∗Cost per Kg of sweet corn = $0.79, ∗∗Control (No fertilizer), ∗∗NR-split (4 bag Urea, 4.5bag SSP, 1.5 bag MOP).

∗∗Cost of production-fertilizer = $ 2063.78, ∗∗FP (3 bags NPK Triple 15, 4bags of Urea), ∗∗RR-NPK (4 bag Urea, 3.5bag SSP, 0.5 bag MOP).

∗∗Cost of NPK triple 15 = $ 30.18/bag, ∗∗NR-NPK (6 bag NPK, 2bag Urea), ∗∗RR-split (5 bag Urea, 4 bag SSP, 33 kg MOP).

∗∗Cost of SSP = $ 34.12/bag, ∗∗ Cost of MOP = $ 61.68 ∗∗Exchange rate = 760 Naira/$.

∗∗Cost of urea = $27.56/bag.

## 4 Discussion

### 4.1 Characterization of maize production

Maize production in Nigeria's mid-belt region is significantly constrained by several factors, including low fertilizer application rates, inappropriate application methods, poor fertilizer quality, and low literacy levels among farmers. These issues lead to inefficient nutrient use, particularly for nitrogen (N) and phosphorus (P), which in turn reduces yields. While potassium (K) use efficiency was generally adequate, the inefficiencies in N and P uptake were likely contributors to yield reductions observed at the study sites. Despite the presence of young and experienced farmers (ages 20–40) with over a decade of farming experience, their limited knowledge of effective fertilizer use is compounded by the inaccessibility of straight fertilizers like SSP and muriate of potash (MOP) to individual farmers. These fertilizers are primarily available only to blending companies through the Nigeria Sovereign Investment Funds (NSIA).

To improve maize yields and nutrient use efficiencies, it is essential to implement more precise fertilizer application methods and timing. Studies by APNI [31] recommend spot application at a depth of 2 cm below the soil surface, while Szulc et al. [40] suggest combining row fertilization with top dressing as the most effective method for maize. However, current farmer practices often deviate from these recommendations, with basal fertilizer typically applied at two WAS and topdressing at four WAS, which significantly reduces yields. It is the general believe of local farmers that the plant are too young for fertilizer application at one WAS. Uduji et al. [41] also highlight that fertilizer usage in Nigeria is well below the FAO-recommended rate of 200 kg ha^−1^, further limiting maize productivity.

### 4.2 Yield and yield attributes

***Plant height:*** The lower plant height (Fig. 1) in the control was mainly due to no fertilizer application. While the NPK blend resulted in taller plants, the RR and split application surprisingly produced highly vigorous shorter plants despite RR's higher fertilizer application rate. This finding challenges the common understanding that higher fertilizer application rates generally result in increased plant height, as reported by Khyber et al. [42], Swe Min [43], Olusegun [44], and Kakar et al. [45]. The shorter but vigorous growth seen with split applications (Fig. 1) aligns with Swe Min [43], who noted that compound fertilizers contributed to greater plant height in rice. Although this study did not explore the reasons behind these observations, it is possible that the timing and method of application, which better match the crop's growth cycle, play a role. Further research is needed to elucidate the underlying mechanisms responsible for the reduced plant height in split applications, even with higher fertilizer rates in maize.

**Tasselling and silking**: The delayed tasselling and silking in the control group (Fig. 2) likely resulted from early crop establishment in fertilized plots, where nutrient availability from the applied fertilizers promoted earlier development. This underscores the importance of fertilizer in achieving timely tasselling and silking in maize. These findings are consistent with the results of Orebo et al. [46], Gheith et al. [47], Sharifi & Namvar [48], and Shrestha et al. [49], who all observed increases in yield and earlier tasselling and silking with higher nitrogen levels in maize fields. The variations in growth among the treatments at the second site may be attributed to early growth disruptions caused by rodent attacks, which disturbed some of the planted seeds during the first week.

***Grain yield:*** The lower grain yield observed in the farmer practice (FP) across both sites (Table 3) can be attributed to several factors, including low fertilizer application rates, inappropriate methods, poor fertilizer quality, low literacy levels, and improper timing of application (SI Table 2). These practices likely led to reduced fertilizer efficiency (Table 4, Table 5, Table 6), ultimately lowering yields. In Site 1, splitting the national recommendation (NR) resulted in an 11.5 % yield increase (Fig. 3) compared to the blend application, whereas in Site 2, it led to an 8 % decrease (Fig. 3). On the other hand, the split application of the regional recommendation (RR) consistently increased yields by 40 % and 12 % in Sites 1 and 2, respectively (Fig. 3). This yield increase with split applications can be attributed to more effective nutrient management, as supported by Sankar et al. [50]. By splitting the fertilizer across three applications during the growing season, plants were able to access nutrients more efficiently (Table 4, Table 5, Table 6), leading to higher yields (Table 3) compared to a single blend application [51,52]. The smaller yield difference in Site 2 (Fig. 3) for both recommendations may be due to inconsistent germination caused by rodent attacks after planting. Nonetheless, the higher yield achieved with the RR compared to the NR (Table 3) is likely due to a combination of higher fertilizer rates and site-specific recommendations that account for regional soil fertility variations [53,54].

***No. of cobs per plant:*** No significant differences Fig. 1were observed in the number of cobs per plant across treatments and sites (Table 3), although the RR treatment showed a slight increase. Similarly, the 100-grain weight remained consistent across all treatments. However, the number of grains per cob was significantly higher for the split application in Site 1 compared to other treatments, while no differences were noted in Site 2 (Table 3). These results indicate that the treatments primarily affected the number of grains per cob rather than cob formation or grain weight, consistent with findings by Băşa et al. [55]. The lower biomass and higher grain yield observed in the RR and split applications (Table 3) are likely due to the shorter plant height (Fig. 1) and more harvest index (Table 4, Table 5, Table 6) which indicate more grain production rather than biomass accumulation. Additionally, the higher protein content in the fertilized plots aligns with the findings of Batyrbek et al. [56], who also reported increased protein content with the application of mineral fertilizers (NPK) compared to unfertilized controls.

### 4.3 Nutrient uptake, efficiencies, and soil properties

***Nitrogen efficiency:*** Given nitrogen's dynamic behavior in the soil—characterized by mobility, leaching, volatilization, and denitrification—maximizing nitrogen uptake is essential for achieving high crop yields. Effective nitrogen uptake relies not only on soil absorption but also on internal processes such as transport, storage, recycling, remobilization, and the plant's growth stage [57]. Bender et al. [28,29] found that the majority of K and N uptake occurs during vegetative growth, with two-thirds of the uptake of N, K, Mg, Mn, B, and Fe occurring before flowering. Similarly, Li et al. [58] reported that a basal fertilizer application ratio of 60.43 %, combined with a topdressing ratio of 39.57 %, effectively enhanced grain filling. This suggests that nitrogen uptake is less significant during grain filling but more rapid in earlier stages. The lower nitrogen use efficiency observed in the FP at both sites (Table 4) highlights the critical importance of timely fertilizer application, as recommended by APNI [59]. The reduced nitrogen uptake and utilization in FP (Table 4) can be attributed to the common practice of late basal fertilizer application at 2 WAS, as well as low and inconsistent application rates (SI Table 2). This underscores the need to adopt improved fertilizer application methods, rather than relying on inefficient practices like surface application and inappropriate timing.

Nitrogen levels in the soil showed slight decreases after harvest (Table 2), likely due to nitrogen's mobility, crop removal, volatilization, and leaching potential [60], as well as the high nitrogen demand for maize [61], which can deplete soil nitrogen reserves [62]. Therefore, when formulating fertilizer recommendations for maize, it is advisable to supply 70–80 % of the crop's nitrogen requirements through a combination of organic amendments, biological or atmospheric fixation, and mineral fertilizers, regardless of the inherent soil nitrogen content. This approach ensures that sufficient nitrogen is available to support optimal maize growth and yield.

Conversely, Bender et al. [28,29] identified two distinct periods of nitrogen uptake: the first occurs between V12 and V18, when yield potential is established (corresponding to when maize has 12 to 18 leaves), and the second during the grain-filling period, when final yield is determined. Additionally, 90 % of the uptake of K, Ca, and Mn occurs before the R2 (reproductive blister) growth stage. Bender et al. [28,29] also recommend a prolonged supply of P, S, Zn, and Cu, while the uptake of N, K, Mg, Mn, B, and Fe mainly occurs during vegetative growth.

**The soil pH**: No significant differences in soil pH were observed after harvest (Table 2), although slight increases of 0.1–0.5 pH units were noted across treatments and sites, with more pronounced changes in the split applications. This variation could be due to the timing of sample collection, with pre-planting samples taken during the dry season and post-harvest samples taken during the rainy season. This reasoning is supported by Vogel et al. [63], who reported that increased soil moisture content can raise pH by up to 1.5 units.

**CEC:** The higher CEC observed with the RR compared to the NR (Table 2) could be attributed to the increased fertilizer application rates in the RR. These higher rates may saturate the soil solution with base cations, thereby enhancing CEC. Supporting this, Demelash et al. [64] demonstrated that combining 8 tons of compost per hectare with 34–10 kg of N-P ha^−1^ can increase soil CEC by 17 %, suggesting a possible synergistic effect of organic and inorganic fertilization on CEC improvement. Similarly, Czarnecki and Düring [65] reported a significant decrease in soil pH, alongside increases in soil carbon content and CEC, following fertilizer application.

***Phosphorus efficiency and Organic matter content:*** Phosphorus availability in tropical soils is influenced by factors such as soil acidity, alkalinity, temperature, and the electrical conductivity of clay surfaces [66]. Nigerian farmers face significant challenges with phosphorus fertilizers, including issues related to fixation and availability. To effectively bridge the maize yield gap, it is essential to ensure that phosphorus fertilizers are accessible to farmers. On average, only 38 % of the total applied P was taken up and assimilated by the crops (Table 5). While the RR demonstrated higher P uptake efficiency (Table 5), the split application proved to be the most efficient method for P uptake (Table 5). Overall, P uptake efficiency was low across all treatments (Table 5), consistent with findings by Bah et al. [67], who noted that P uptake efficiency is generally lower with mineral fertilizers compared to green manure in tropical soils. Incorporating poultry manure alongside inorganic fertilizers could be a more effective strategy for improving P utilization, as suggested by Zafar et al. [68]. The unutilized P is likely to become bound to clay minerals or fixed in the soil, potentially serving as a nutrient source for future crops. The higher phosphorus application rates in the RR led to a slight increase in available P levels after harvest compared to farmer practices and the NR (Table 2). This suggests that RR is more effective in maintaining soil P availability for subsequent crops. The low organic matter content in the soil (Table 2) is likely due to its loamy sand texture (Table 2), which typically has less capacity to retain organic matter. Hartati et al. [69] noted that soil organic matter tends to increase gradually with higher clay content. In Site 1, organic matter content remained relatively stable after harvest, while in Site 2, it decreased (Table 2). To enhance and maintain soil organic matter levels, implementing conservation tillage practices such as zero-tillage and retaining plant residues [70], along with incorporating organic manure [71], could be effective strategies.

**Base Cations**: After harvest, exchangeable potassium (K^+^), calcium (Ca^2+^), and magnesium (Mg^2+^) levels showed slight increases, with the RR treatment showing the most pronounced effect (Table 2). This could be attributed to increased base cation saturation in the soil solution resulting from the higher fertilizer application rates in the RR. Aliyu et al. [7] reported that soils in Nigeria's mid-belt (Guinea savanna) are relatively low in total nitrogen (0.69 g kg⁻^1^) and available phosphorus (3.79 mg kg⁻^1^), as shown by positive yield responses to N and P in nutrient omission trials. However, the response to potassium (0.50 cmolc kg⁻^1^) was negative, indicating that K levels were above the critical threshold. The study also reported a negative response to several macro- and micro-nutrients (Ca, Mg, S, B, Zn, and Cu), suggesting that these nutrients are not significantly limiting maize production in the region. Conversely, a positive response to micronutrients (S, B, Zn, and Cu) was observed, though this contradicts the negative response reported by Shehu et al. [72]. While the observed changes in this study may not be statistically significant, long-term effective fertilizer management could provide a sustainable means of maintaining soil fertility in the study area.

***Potassium efficiencies:*** Potassium uptake efficiency was generally high across all treatments (Table 6), which may be attributed to the findings of Ahmed et al. (2010) that kaolinite clay minerals, such as those in ultisols and oxisols found in tropical climates like Nigeria, are rich in potassium. Although potassium availability is not typically a major issue in tropical soils, its uptake and efficiency can be compromised by leaching, particularly in areas with high rainfall intensity (Ahmed et al., 2010). In highly weathered soils with low pH, elevated levels of Al^3+^ and Fe^2+^ can displace base cations, including K^+^, from soil exchange complexes, making it difficult for potassium to remain in the soil solution [73]. Nutrients not absorbed by crops may not be entirely lost but can become immobilized within the soil organic matter pool.

**Agronomic efficiency:** In this study, neither blend nor split fertilizer applications resulted in significant variations in AE for maize. The lower AE observed in the farmer practice (FP) compared to other treatments is likely due to the common practice among farmers of surface-applying fertilizer, where nutrient uptake is less efficient. The high coefficient of variation (CV) values, ranging from 57.55 % to 71.01 %, indicate considerable variability in the data, suggesting that longer-term studies may be necessary to draw more definitive conclusions about the impact of different fertilizer blends on AE.

### 4.4 Economic analysis

The cost of fertilizer varied among treatments (Table 7), with split applications, despite being more expensive than NPK blends due to scarcity, ultimately resulted in a greater return on investment (ROI) than NPK blends. This highlights the potential economic benefits of adopting more precise and efficient fertilizer management strategies. While the specific cost differences and ROI values may vary depending on individual circumstances, the underlying message remains clear: effective fertilizer management through split applications can significantly enhance economic returns for maize farmers in mid-beat Nigeria.

#### 4.4.1 Policy implications, constraints, and futuristic scope

This study demonstrates the effectiveness of straight versus blended fertilizers, offering data-driven recommendations for shaping future fertilizer subsidy programs. Emphasizing the need for precise nutrient management tailored to regional soil and crop requirements, the findings can help policymakers develop guidelines and support systems for optimal fertilizer application. This, in turn, would boost maize yields and address the gap between production and demand. It is important to modernize fertilizer blending technologies to prevent particle segregation and enhance crop productivity. It advocates for ensuring that straight fertilizers (MOP, DAP, SSP, TSP) are readily available to farmers, not just blending companies.

Variability in soil quality and farming practices across diverse geographical areas limits the study's scope and affects the generalizability of the findings. Future research should explore the long-term impacts of different fertilizer types on soil health and crop productivity. Advancements in precision agriculture technologies, such as remote sensing and soil nutrient mapping, could enhance the accuracy of fertilizer application recommendations. Expanding the scope to include other staple crops and regions within Nigeria is essential for a comprehensive understanding of optimal fertilizer practices nationwide. Although young and experienced farmers dominate agriculture in the area, their limited literacy constrains their understanding of effective fertilizer use. To address these challenges, the following recommendations are made:

● ***Educational program for farmers:*** With many farmers having limited formal education, training programs are crucial to enhance their knowledge and skills, particularly in good agronomic practices and context-specific solutions. These programs should focus on the principles of 4R stewardship (right source, right rate, right time, and right place), the importance of nitrogen management, optimal application timing, and the ability to assess the quality of different fertilizer products.
● ***Promote Encouragement of basal fertilizer application:*** There is a noticeable gap in the timely application of basal fertilizers, with many farmers not applying them within the recommended one week after sowing. Farmers need education on the benefits of timely basal fertilizer application and how it can significantly impact crop yields.
● ***Diversification in fertilizer application:*** To ensure a balanced nutrient supply for crops, farmers should be encouraged to use a broader range of fertilizers. Government agencies should facilitate access to and promote the use of straight fertilizers like single superphosphate, urea, and muriate of potash, which are essential for maximizing crop growth and yield.
● ***Optimization of fertilizer practices:*** Continuous monitoring of crop responses to different fertilizer applications will allow farmers to refine their methods for improved outcomes. The practice of split applications based on regional recommendations is advised due to its superior return on investment compared to national recommendations despite potentially higher fertilizer costs.

## 5 Conclusions

This study demonstrates that optimizing fertilizer application methods, particularly through split applications of straight fertilizers, can significantly improve maize yield, nutrient use efficiency, and economic returns in Nigeria's mid-belt region. Despite the challenges faced by farmers, such as low literacy levels and limited access to high-quality fertilizers, adopting more precise nutrient management strategies has the potential to overcome these barriers and enhance overall productivity. Split application of regional recommendations (119:38:20 kg ha⁻^1^ of N, P, and K) using straight fertilizers results in higher maize yields and improved nutrient efficiency compared to NPK blends, proving effective for optimal maize production in Nigeria's mid-belt region. Additionally, the use of straight fertilizers is associated with decreased plant height and biomass yield. The major yield-limiting factors in this region include low fertilizer application rates, improper methods, poor fertilizer quality, low literacy levels, and inefficient nutrient use.

## Funding

This research was funded by OCP Africa.

## Data availability statement

The data for this research is available and will be provided upon request.

## CRediT authorship contribution statement

**Kehinde Ojeniyi:** Writing – original draft, Visualization, Methodology, Investigation, Formal analysis, Data curation, Conceptualization. **Chirinda Ngonidzashe:** Writing – review & editing, Supervision. **Krishna Devkota:** Writing – review & editing, Supervision. **Donald Madukwe:** Writing – review & editing, Supervision, Funding acquisition.

## Declaration of competing interest

The authors declare the following financial interests/personal relationships which may be considered as potential competing interests:Ojeniyi Kehinde Afeez reports financial support was provided by OCP Africa. Chirinda Ngonidzashe reports a relationship with University of Mohammed VI Polytechnic, UM6P Morocco that includes: employment. Krishna Devkota reports a relationship with International Center for Agricultural Research in the Dry Areas that includes: employment. Donald Madukwe reports a relationship with OCP Africa that includes: employment. If there are other authors, they declare that they have no known competing financial interests or personal relationships that could have appeared to influence the work reported in this paper.
